# Impacts on knowledge and testing on HIV in waves of Mozambique surveys with Bayes estimates

**DOI:** 10.1371/journal.pone.0244563

**Published:** 2020-12-29

**Authors:** Ziwei Chen, Adriana Dornelles, Di Fang, Jeffrey R. Wilson

**Affiliations:** 1 School of Mathematics and Statistics, Arizona State University, Tempe, Arizona, United States of America; 2 Department of Economics, Arizona State University, Tempe, Arizona, United States of America; 3 Agribusiness Department, University of Arkansas, Fayetteville, Arkansas, United State of America; 4 Department of Economics, Arizona State University, Tempe, Arizona, United States of America; Sheffield Hallam University, UNITED KINGDOM

## Abstract

**Background:**

It is well known that it is more reliable to investigate the effects of several covariates simultaneously rather than one at time. Similarly, it is more informative to model responses simultaneously, as more often than not, the multiple responses from the same subject are correlated. This is particularly true in the analysis of Mozambique survey data from 2009 and 2018.

**Method:**

A multiple response predictive model for testing positive for HIV and having sufficient HIV knowledge is modeled to 2009 and 2018 survey data with the use of Bayes estimates. These data are obtained through a hierarchical data structure. The model allows one to address the change in the response to HIV, as it relates to morbidity and to HIV knowledge in Mozambique in the fight against the disease in the last decade.

**Results:**

A more affluent resident is more likely to test positive, more likely to be more knowledgeable about the disease. Whereas, individuals practicing the Islam faith are less likely to test positive but also less likely to be knowledgeable about the disease. Education, while still a factor, has declined in its impact on testing positive for HIV or being knowledgeable about HIV. Females are more likely to test positive but more likely to be knowledgeable about the disease than men. The rate of impact of affluence on knowledge has increased in the past decade. Marital status (cohabitating or married) showed no impact on the knowledge of the disease. Age had no impact on knowledge suggesting that the message is getting to resident.

**Conclusions:**

A joint Bayes modeling of correlated binary (testing positive and knowledge about the disease) responses, while accounting for the hierarchy of the data collection, presents an opportunity to extract the extra variation before allocating the variation on the responses as the due of the covariates. The fight against HIV in Mozambique seems to be succeeding. Some knowledge is common among all ages, and Islam religion has a positive effect. While education still shows an influence on the binary responses, it has declined over the last decade.

## Introduction

It is common in national health research surveys to use the results from the analysis of data to advance research policy. Survey results help to suggest policy priorities. In the research of HIV, [1] found that testing benefits those who test positive, allowing them to receive treatment but the benefits for those who test negative is somewhat controversial. They suggest that HIV testing may increase knowledge and result in a reductions in sexual risk even when results are negative. Ongoing post-test education and support are needed to maintain their HIV-negative status. Testing benefits those who test positive, allowing them to receive antiretroviral treatment. HIV testing is an effective strategy to reduce the spread of HIV. It makes people aware of their HIV status and promote safer sex behaviors [2]. The knowledge of HIV transmission and the opportunity to prevent are necessary for the reduction of sexual risk behaviors. Transmission of HIV in sub-Saharan Africa nations continues to be high due to a large proportion of individuals living with undiagnosed HIV. A multitude of factors including the patient’s knowledge and beliefs about HIV influence HIV testing, [3].

The Demographic and Health Survey (DHS) is conducted in over 90 nations globally to obtain representative data on population health, nutrition, and HIV/AIDS (DHS, 2018). Data from these surveys are analyzed to identify trends and are used to advance global research agendas and national programs and policy [4,5]. These national health surveys are used to generate information that is critical in describing national and regional trends and identifying gaps in knowledge. However, suboptimal analytic practices threaten the evidence base used for programmatic and policy decisions. Although national surveys capture multiple outcomes of interest, these outcomes are often modeled one-at-a-time, and common causes are not realized.

Mozambique is an example of a nation in sub-Saharan Africa that is severely impacted by the HIV/AIDS epidemic. The disease is one of the single largest global health priorities of the past two decades, with $562.6 billion spent globally between 2000 and 2015 (Global Burden of Disease Health Financing Collaborator Network, 2018). Innumerable analyses have characterized the HIV/AIDS epidemic and its drivers within and across contexts [6,7].

A hierarchical structure is common in national survey and usually results in correlated observations that are often overlooked. This sort of omission of correlation leads to incorrect conclusions due to the incorrect standard errors that are reported [8,9]. As such, joint modeling of correlated binary responses while accounting for the hierarchical structure of the data through random effects is now a reality with the advent of many statistical programs, including PROC MCMC in the release of SAS 2017.

As the data for the national surveys conducted in Mozambique for 2009 and for 2018 became available, it is of interest to model one’s knowledge of the disease and testing positive. These two measures relate to the core of understanding and combating the disease. The threat of the HIV disease still hovers over the sub-Saharan Africa nation, so importantly, these data are used to measure the strength and the impact of the system over the last decade. A simultaneous model for probability of one’s knowledge and probability of testing positive result presents regression parameter estimates with Bayes principles. The data are available on the web under Mozambique health data. This research focuses on the hierarchical structure of the data and the gain in the simultaneous modelling of correlated binary responses.

## Methods

### Mozambique survey data

There are 306 clusters (primary units) in 2018 and 270 clusters (primary units) in 2009 distributed and sampled across Mozambique’s 11 provinces. These data consist of 5,798 households in 2018 (secondary units) and 4,988 households in 2009 (secondary units) eligible for sampling. Men and women aged 15–64 living in these households are at the observational level and eligible to participate by giving blood samples. This resulted in 10,648 adult participants in 2018 and 8,818 adult participants in 2009.

#### Outcomes of interest

A binary variable one’s knowledge of HIV is measured by one’s exposure to a culmination of sources where information is delivered including “Community meetings”, “school”, “hospitals” or “Community health worker” provides one outcome. Also the result of a blood test administered to all survey respondents provides another binary outcome of interest.

#### Covariates

The covariates used in the study include the binary variables, “have electricity” and “have a refrigerator,” while the categorical variables include a wealth index on an ordinal scale of poorest, poorer, middle, richer, and richest. Religions of different types are: Catholic, 6, Zion, Evangelical, Anglican, Protestant, and no religion. However, as Islam is the most popular religion it is of interest to look at Islam versus the others, thereby making use of a binary variable in religion. Marital status had several categories: never married, married, living together, widowed, and divorced. “Living together” and “married” are combined to present a binary variable. Work had several categories: did not have a job, had a job in past year, had a job currently, had a job but was on leave for seven days. The variables, “had a job currently” or “had a job in the past year” or “had a job but was on leave for seven days” are combined and represented by a binary variable. The continuous measures are education in years and age.

#### Prior information

Previously analyzed information obtained from the 2009 survey in Mozambique [10] is used as priors in the analysis, as shown in Table 1. The information is incorporated through the distributional form of the parameters along with the present likelihood of the latest data.

**Table 1:** Regression parameter estimates for analysis of 2009 data.

Variable	Knowledge	Std. Error	Testing +ve	Std. Error
**Intercept**	0.940**	0.120	-3.080**	0.151
**Electricity**	0.451**	0.116	-0.044	0.105
**Refrigerator**	0.457**	0.149	-0.471**	0.116
**Wealth**	0.083	0.077	0.817**	0.091
**Education**	0.151**	0.011	0.018	0.011
**Age**	-0.008**	0.002	0.011**	0.003
**Religion**	-0.125	0.079	-0.318	0.109
**Cohabitants**	-0.008	0.055	-0.101	0.067
**Female**	-0.054	0.059	0.474**	0.071

** denotes significant values.

#### Hierarchical model

The survey data are obtained through a hierarchical structure. The individuals are nested within households and the households are nested within clusters. There are correlations at the cluster level, and there are correlations at the households within the clusters level. These extra levels of correlations are accounted for in the analysis, usually through the use of random effects. The variation caused by these hierarchical structures are different, therefore the variation of testing positive differs from the variation in knowledge both giving rise to modeling correlated responses.

The modeling of binary outcomes often makes use of a logistic regression model as a member of the class of generalized linear models. However, as the hierarchical data present a structure that destroys the independence assumption, one must resort to a hierarchical generalized linear mixed model to account for the intraclass correlation at the different levels of the hierarchical structure. This correlation is often accounted for through the use of random effects at various level [9]. When modeling a binary response in a hierarchical structure, and then at the observational level, one assumes the dichotomous outcome comes from an unknown latent continuous variable with a level-1 residual that follows a logistic distribution with a mean of 0 and a variance of 3.29 [11]. Therefore, 3.29 is used as the level-1 error variance in calculating the ICC.

Thus, modeling one response, a generalized linear mixed model with the clusters and the households incorporated as random effects to model the contribution due to households and due to clusters respectively,

$$logit(p_{response})=\log\left[\frac{p_{response}}{1-p_{response}}\right]=\beta_{0}+\beta_{1}X_{i1hc}+\cdots +\beta_{P}X_{iPhc}+u_{oc}+u_{ohc}$$

where p_response_ is the probability of a response (testing positive or some knowledgeable about HIV) for the i^th^ resident within the h^th^ household within the c^th^ cluster, β_i_ is the regression coefficient associated with the predictor X_ihc_ for i = 1,2, …, n_hc_; are the covariates associated with the h^th^ household within that c^th^ cluster, and a random effect household *h* = 1, 2, …, n_c_; and cluster c = 1, …, n; the random intercept u_oc_ measures the unobserved variance attributable to the c^th^ cluster, the random intercept u_ohc_ measures difference of household level h within cluster c. These two random effects are assumed to be normally distributed with (clusters) $u_{oc}∼N(0,{\sigma^{2}}_{u_{c}})$ and (households within cluster) $u_{ohc}∼N(0,{\sigma^{2}}_{u_{hc}})$. Further, we assume that the covariance of the random effects, $\sigma_{u_{oc},u_{ohc}}$ is zero. Households as random effects represent the differences on the resident’s response attributable to households, but were not captured by any of the covariates. Similarly, clusters random effects represent the differences on the resident’s response attributable to the clusters, but were not captured by any of the covariates.

Modeling each of these outcomes separately will ignore the collective impact that some of these predictors nay have on a response through the correlation between the two responses. However, a simultaneous modeling of these outcomes will help identify any such predictor. Knowing that a predictor has such multiple effect will help policy makers with identifying optimal solutions [12].

#### Simultaneous models

In public health research, as well as research in other disciplines, it is common for subjects to provide information through a range of responses with a set of covariates. However, modeling several responses is more informative to public health officials and decision makers, as it allows one to properly evaluate the interplay of the responses. Thus, it is advantageous to do joint modeling when the opportunity arises [13]. In this study, and as previous research dictates, the simultaneous modeling of testing positive and some knowledge of the disease are important to policy makers in their efforts to contain the disease [14,15].

The two simultaneous correlated binary outcomes Y_qihc_, *q* = 1, 2, denoting the i^th^ individual on the h^th^ household member of the c^th^ cluster for *h* = 1,…..*n*_*c*_, and *c* = 1,…..307. A joint model of these correlated binary outcomes f(Y_1hc_, Y_2hc_,), for *q* = 1, for test positive and *q* = 2, for knowledgeable about HIV, follow a joint Bernoulli distribution with mean p_1hc_ and random effects u_oc_ for clusters and random effects u_ohc_ for households,

$$logit(p_{qhc})={\beta_{00}}^{q}+{\beta_{1}}^{q}x_{1hc}+\cdots +{\beta_{p}}^{q}x_{phc}+u_{oc}+u_{ohc}.$$

### Bayesian hierarchical modeling

In the analysis of the 2009 Mozambique data, the joint distribution of probability of testing positive and probability of having one’s knowledge about the disease produced some sparseness in the results. Less than 1% tested positive but had no knowledge of the disease, as shown in Table 2. Such sparseness leads one to consider an application of Bayes’ principles in estimating the coefficients in the model.

**Table 2:** Distribution of responses for joint binary responses.

Testing +ve	Knowledgeable	Frequency	%
0	0	1245	11.69
1	0	99	0.93
0	1	7807	73.32
1	1	1497	14.06

The Bayes’ principle requires a prior probability distribution on the parameters, Table 1. They were obtained from an analysis of the 2009 survey data and they provides prior knowledge of the parameters [12]. This prior information, combined with the likelihood of the observations and the distribution of the random effects, resulted in a posterior distribution. Through the use of Markov Chain Monte Carlo (MCMC), one can obtain posterior information about the regression parameters. While there are several methods to generate the posterior samples, including Gibbs Sampling and Metropolis-Hasting sampler, the deliberation samples are drawn directly from the full conditional distribution by using standard random number generators.

$$logit(p_{qhc}+\theta_{oc}+\theta_{ohc})={\beta_{00}}^{q}+{\beta_{1}}^{q}x_{1hc}+\cdots +{\beta_{p}}^{q}x_{phc}+\theta_{oc}+\theta_{ohc}.$$

The simultaneous responses with a hierarchical structure consist of binary responses, so that the probability of a response q (r = 1,2) denoted by p_qhc_ and the logit(p_qhc_+θ_oc_+θ_ohc_) where θ_oc_ denotes the household effects and θ_ohc_ denotes cluster effect. The Bayes hierarchical mixture model is summarized in Table 3. In Table 3, the parameters and their distribution at various levels of the hierarchy are given.

**Table 3:** Hierarchical structure n Bayes model specifications.

Level	Mean Prior	Type	Variance Priors	Type
Individual	$Y_{i}∼D(u_{ihc},\sigma_{hc}^{2})$	Random effects	- -	- -
Household	$u_{ihc}∼D(u_{hc},\sigma_{h}^{2})$	Random effects	$\sigma_{hc}^{2}∼IG(\alpha_{o},\beta_{o})$	hyperparameter
Cluster	$u_{hc}∼D(u_{c},\sigma_{hc}^{2})$	Hyper-hyperparameter	$\sigma_{h}^{2}∼IG(\alpha_{oo},\beta_{oo})$	Hyper-hyperparameter

## Results

In the 2018 survey data, approximately 60% of respondents are female, while in 2009 there are 57% are female. There are 33% respondents who are living with a partner in 2018, in contrast to 58.71% in 2009. A summary of these results is presented in Table 4.

**Table 4:** Mean of variables in 2009 versus 2018.

Variable	2009	2018
**Females**	57%	60%
**Living with partner**	58.71%	33%
**Average age**	33 years	31 years
**Years of education**	4 years	5 years
**Muslim**	18.13%	16.56%
**Refrigerator**	15.16%	28.02%
**Electricity**	24.5%	39.7%
**Poor**	48.72%	54.25%

There are 1166 (13.22%) respondents who tested positive in 2009, and 1596 (14.99%) who tested positive in 2018. Of the respondents, 76.82% reported some knowledge of HIV in 2009, and 87.38% in 2018, as shown in Table 5.

**Table 5:** Joint responses to two binary responses.

Response		Distribution in 2009		Distribution in 2018	
Test +ve	Knowledge	Frequency	Percent	Frequency	Percent
0	0	1839	20.86	1245	11.69
1	0	205	2.32	99	0.93
0	1	5813	65.92	7807	73.32
1	1	961	10.90	1497	14.06

The survey is designed to account for the differences at the cluster level and at the household level. The data suggest that 0.736/(0.736+0.443+3.29) = 16.47% of the variation in test results are due to cluster effects. While 0.443/(0.736+0.443+3.29) = 9.92% of the variation are due to households within cluster effects (Table 5). However, in 2018, the clusters differences accounts for 13% and households accounts for 11.70%. In both surveys, the data structure accounted for about 25% of the variation. Moreover, the random effects (in clusters and households within clusters) are significant, as shown in Table 6.

**Table 6:** Covariance parameter estimates due to clusters and households.

	Blood Test in 2009		Blood Test in 2018	
Subject	Estimate	Standard Error	Estimate	Standard Error
**cluster**	0.736	0.101	0.568	0.074
**house(cluster)**	0.443	0.087	0.511	0.077

A simultaneous Bayes model with interaction effects is fit to the 2019 data with use of the prior information in the parameters obtained from the 2009 survey. The interaction effects serve to measure the covariate effects in 2009, as compared to a decade later in 2018.

Residents equipped with a refrigerator are 1.349 times more likely to be knowledgeable about HIV/AIDS (95% HPD Interval [1.135, 1.587]), as opposed to those who are not equipped with a refrigerator. The more affluent residents are 1.265 times more likely to be knowledgeable about the disease (95% HPD Interval [1.108, 1.443]). A resident with an extra year of education has 1.196 times more likely to be knowledgeable about HIV/AIDS, (95% HPD Interval [1.172, 1.218]). Non-Muslims are (1/0.773 =) 1.29 times more likely to be knowledgeable of the disease, (95% HPD Interval [1.16, 1.43]). Females are 1.398 times more likely to be knowledgeable about the disease (95% HPD Interval [1.285, 1.516]). As for the effect change over the decade, more affluent residents continued to be more knowledgeable (95% HPD Interval [1.146, 1.718]). While cohabitating residents are less knowledgeable, the effect has steadily increased over the last decade (95% HPD Interval [1.573, 2.255]). While having more education continues to have a positive effect on knowledge of HIV, the effect of education has declined over the decade (95% HPD Interval [0.906, 0.957]), as shown in Table 6.

The more affluent residents are 2.275 times more likely to test positive (95% HPD Interval [2.059, 2.527]). Women are 1.675 times more likely to test positive (95% HPD Interval [1.531, 1.831]). Older residents are 1.021 times likely to test positive (95% HPD Interval [1.018, 1.024]). Those residents practicing Islam are 0.671 times less likely to test positive (95% HPD Interval [0.590, 0.771]). Residents with an extra year of education are 1.024 times more likely to test positive (95% HPD Interval [1.005, 1.042]). While more educated residents are more likely to test positive, that tendency is declining (95% HPD Interval [0.930, 0.973]), (Table 7).

**Table 7:** HPD interval estimates for odds for testing positive and knowledge.

Variable	Knowledge	95% HPD Interval	Test +ve	95% HPD Interval
**Intercept**	1.446	(1.270, 1.652)	0.032	(0.026, 0.038)
**Electricity**	1.349	(1.135, 1.587)	--	**- -**
**refrigerator**	1.262	(1.051, 1.547)	0.687	(0.608, 0.770)
**Affordable**	1.265	(1.108, 1.443)	2.275	(2.059, 2.527)
**Education**	1.196	(1.172, 1.218)	1.024	(1.005, 1.042)
**Age**			1.021	(1.018, 1.024)
**Muslim**	0.773	(0.699, 0.860)	0.671	(0.590, 0.771)
**Cohabitants**	0.937	(0.832, 1.059)	--	**- -**
**Female**	1.398	(1.285, 1.516)	1.675	(1.531, 1.831)
**Survey year**	1.614	(1.392, 1.883)	1.533	(1.358, 1.770)
Education * Year	0.931	(0.906, 0.957)	0.951	(0.930, 0.973)
Affordable * year	1.418	(1.146, 1.718)	--	**- -**
Cohabitants*year	1.889	(1.573, 2.255)	--	**- -**

## Discussion

Modeling simultaneous responses is a cost-saving approach as it allows researchers to address the interplay of responses and extra variation. It allows covariates to compete for their effects on responses. More importantly, the simultaneous modeling of binary responses on hierarchical structure data of future surveys provides policymakers and decision makers with information on which to base allocation of resources at a time when funding is in scarce commodity.

The Mozambique household survey data are a hierarchical structure, based on responses from households, and households are taken from the clusters. Such a structure consists of multiple sources of variation: variation due to the clustering of observations from the same household, variation between households taken from clusters and variation among the effects due to spillover. Thus any modeling of those data must include the multiple types of variation.

The results provide evidence that one’s HIV knowledge is associated with those who report testing negative for HIV, Muslim and those with more education are necessary for a reduction of sexual risk behaviors. There is evidence that supports for an initiative to continue to provide prevention education even to those who test negative for HIV. Ongoing education is necessary to maintain low HIV-negative status. It is not always clear at what age respondents get education and knowledge on HIV.

As policy makers study these results, it is clear that females, non-Muslims, those with more years of education, older residents, and those who are more affluent are more likely to test positive. Furthermore, females who are more affluent, more educated, non-Muslim are more likely to be knowledgeable. The simultaneous model reveals that affluence, education, religion, and gender had similar effects on testing positive and being knowledgeability about the disease. While having more knowledge is desirable, it seems to increase the chances of testing positive. We had no way of measuring the type of knowledge and we had no way of depicting when that knowledge was acquired.

Greater affluence seems to increase the likelihood of testing positive. It appears that knowledge of HIV is reaching all ages; however, older residents are more likely to test positive. This result may be due to the fact that they have lived longer and thus had more chances to become infected. Religious practices seem to have an important effect, while cohabitating had no effect in 2009 or 2018. Based on the analysis of the results from the Mozambique national survey data, the spread of the disease has remained relatively stable over the past decade, and greater knowledge about HIV among all ages of the population may be one reason for this stability.

Change have occurred in Mozambique in the last decade to impact the spread of the HIV disease. The country is gaining in their fight to minimize the spread of the disease.

## Supporting information

S1 Appendix (DOCX)

## References

[1] Scott-Sheldon LA, Carey MP, Carey KB, et al HIV testing is associated with increased knowledge and reductions in sexual risk behaviours among men in Cape Town, South Africa. *Afr J AIDS Res*. 2013;12(4):195–201. 10.2989/16085906.2013.863219 25871481 PMC4520431

[2] HIV voluntary counseling and testing and behavioral risk reduction in developing countries: a meta-analysis, 1990–2005. Denison JA, O'Reilly KR, Schmid GP, Kennedy CE, *Sweat MD AIDS Behav*. 2008 5; 12(3):363–73. 10.1007/s10461-007-9349-x 18161018

[3] Ryan S., Hahn E., Rao A. et al The impact of HIV knowledge and attitudes on HIV testing acceptance among patients in an emergency department in the Eastern Cape, South Africa. *BMC Public Health* 20, 1066 (2020). 10.1186/s12889-020-09170-x 32631297 PMC7339484

[4] Lawn SD, Kranzer K and Wood R. Antiretroviral therapy for control of the HIV-associated tuberculosis epidemic in resource limited settings. *Clinics in Chest Medicine*. 2009; 30(4):685–699. 10.1016/j.ccm.2009.08.010 19925961 PMC2887494

[5] Mbachu C, Okoli C, Onwujekwe O, Enabulele F. Willingness to pay for antiretroviral drugs among HIV and AIDS clients in south-east Nigeria. *Health Expect*. 2017; 21(1):270–278. 10.1111/hex.12612 28805985 PMC5750729

[6] Mayer KH, Beyrer C. HIV Epidemiology update and transmission factors: Risks and Risk Contexts-16^th^ Int. AIDS Conference Epidemiology Plenary. *Clinical Infectious Diseases*. 2007; 44(7): 981–987. 10.1086/512371 17342654

[7] Pradhan NS, Su Y, Fu Y, Zhang L, Yang Y. Analyzing the effectiveness of policy implementation at the local level: A case study of management of the 2009–2010.

[8] Collett D. *Modelling binary data*. Chapman and Hall; 1991.

[9] Irimata KM, Wilson JR. Identifying intraclass correlations necessitating hierarchical modeling. *Journal of Applied Statistics*. 2017; 16:1–16.

[10] Fang D, Sun R, Wilson JR. Joint modeling of correlated binary outcomes: The case of contraceptive use and HIV knowledge in Bangladesh. *PLOS ONE*. 2018; 13(1):e0190917 10.1371/journal.pone.0190917 29351328 PMC5774700

[11] Ene M, Leighton EA, Blue GL, Bell BA. Multilevel models for categorical data using SAS® PROC GLIMMIX: The basics. *SAS Global Forum 2015 Proceedings*; 2015.

[12] Fang D., Lang A. & Wilson J.R. HIV survey in Mozambique: analysis with simultaneous model in contrast to separate hierarchical models. *Arch Public Health* 78, 70 (2020). 10.1186/s13690-020-00453-8 32765847 PMC7395358

[13] Vazquez-Arreola E., Irimata K and Wilson JR (2020) Common errors of interpretation in biostatistics. *Biostatistics and Epidemiology* 4:1,238–246, 10.1080/24709360.2020.1790085

[14] Gueorguieva RV, Agresti A. A correlated probit model for joint modeling of clustered binary and continuous responses. J Am Stat Assoc. 2001;96: 1102–12.

[15] Wilson JR and Lorenz KA Modeling binary correlated responses using SAS, SPSS and R (2015) Springer.

