# Supplementary material for: Impacts on knowledge and testing on HIV in waves of Mozambique surveys with Bayes estimates
Source: PLoS One. 2020 Dec 29;15(12):e0244563. doi: 10.1371/journal.pone.0244563 (PMC7771863; doi:10.1371/journal.pone.0244563)
Supplement: S1 Appendix — (DOCX) [file pone.0244563.s001.docx]

**appendix**

dm "log;clear;output;clear";

options ps=**100** ls=**78** formdlim='*' nodate nonumber;

**proc** **import** datafile = '/folders/myfolders/Mozambique2009.xls'

out = work.Moz2019

dbms = XLS

;

**run**;

****************** merged ************************************;

**proc** **import** datafile = '/folders/myfolders/Mozmerged 2018&2009 clean.xlsx'

out = work.Mzmhh

dbms = XLSX

;

**run**;

**data** Mozmerged;

set work.Mzmhh;

**run**;

**quit**;

****************** interaction ************************************;

*********work, wealth,education,living together and year;

**data** Mozmerged;

set Mozmerged;

yeareducation=year*eduyrs;

yearwork=year*work;

yearwealth=year*wealth;

yearlivtog=year*mar;

**run**;

**proc** **mcmc** data=Mozmerged seed=**5225** nmc=**50000** thin=**20** plot=trace diag=GEWEKE DIC STATISTICS;

parms B0_k

ELCT_k /*ELECTRICITY*/

FGE_k /*fridge*/

WEA_k /*WEALTH INDEX*/

EDU_k /*education years, do it as continuous variable*/

AGE_k /*age, do it as continuous variable*/

R_k /*RELIGION*/

M_k /*marital status*/

W_k /*WORK12*/

G_k /*gender*/

year_k

yredu_k

yrwea_k

yrmar_k

yrwork_k

B0_h

ELCT_h /*ELECTRICITY*/

FGE_h /*fridge*/

WEA_h /*WEALTH INDEX*/

EDU_h /*education years, do it as continuous variable*/

AGE_h /*age, do it as continuous variable*/

R_h /*RELIGION*/

M_h /*marital status*/

W_h /*WORK12*/

G_h /*gender*/

year_h

yredu_h

yrwea_h

yrmar_h

yrwork_h

H2

C2 ; /*random effects */

prior B0_k: ~ normal(**0.9404**, sd=**1**);

prior ELCT_k:~ normal(**0.4512**,sd=**1**);

prior FGE_k: ~ normal(**0.4574**, sd=**1**);

prior WEA_k : ~ normal(**0.0830**, sd=**1**);

prior EDU_k : ~ normal(**0.1505**, sd=**1**);

prior AGE_k : ~ normal(-**0.008**,sd=**1**);

prior R_k : ~ normal(-**0.1247**, sd=**1**);

prior M_k : ~ normal(-**0.00829**, sd=**1**);

prior W_k : ~ normal(**0.0685**,sd=**1**);

prior G_k : ~ normal(-**0.0544**,sd=**1**);

prior year_k: ~ normal(**0**,sd=**1e6**);

prior yredu_k:~ normal(**0**,sd=**1e6**);

prior yrwea_k:~ normal(**0**,sd=**1e6**);

prior yrwork_k:~ normal(**0**,sd=**1e6**);

prior yrmar_k:~ normal(**0**,sd=**1e6**);

prior B0_h: ~ normal(-**3.0795**, sd=**1**);

prior ELCT_h:~ normal(-**0.0443**,sd=**1**);

prior FGE_h: ~ normal(-**0.4713**, sd=**1**);

prior WEA_h : ~ normal(**0.8166**, sd=**1**);

prior EDU_h : ~ normal(**0.0177**, sd=**1**);

prior AGE_h : ~ normal(**0.0109**, sd=**1**);

prior R_h : ~ normal(-**0.3183**, sd=**1**);

prior M_h : ~ normal(-**0.1011**, sd=**1**);

prior W_h : ~ normal(**0.0826**,sd=**1**);

prior G_h : ~ normal(**0.4744**,sd=**1**);

prior year_h : ~ normal(**0**,sd=**1e6**);

prior yredu_h:~ normal(**0**,sd=**1e6**);

prior yrwea_h:~ normal(**0**,sd=**1e6**);

prior yrwork_h:~ normal(**0**,sd=**1e6**);

prior yrmar_h:~ normal(**0**,sd=**1e6**);

prior C2 ~ igamma(**34.1264**, scale =**6.8638**);

random clst~ n(**0**, var=C2) subject=cluster;

prior H2 ~ igamma(**7.9176**, scale =**0.0266**);

random household~ n(clst, var=H2) subject= hh;

Mu1=B0_k

+ ELCT_k*electricity

+ FGE_k*fridge

+ WEA_k*wealth

+ EDU_k*eduyrs

+ AGE_k*age

+ R_k* reli_islc

+ M_k*mar

+ W_k*work

+ G_k*gender

+ year_k*year

+yredu_k*yeareducation

+yrwea_k*yearwealth

+yrmar_k*yearlivtog

+yrwork_k*yearwork

+ household ;

p_k = logistic(Mu1);

model knowtest ~ binary(p_k);

Mu2=B0_h

+ ELCT_h*electricity

+ FGE_h*fridge

+ WEA_h*wealth

+ EDU_h*eduyrs

+ AGE_h*age

+ R_h* reli_islc

+ M_h*mar

+ W_h*work

+ G_h*gender

+ year_h*year

+yredu_h*yeareducation

+yrwea_h*yearwealth

+yrmar_h*yearlivtog

+yrwork_h*yearwork

+ household ;

p_h = logistic(Mu2);

model HIV03 ~ binary(p_h);

preddist outpred=predout nsim=**10**;

**run**;

**proc** **transpose** data=predout; **run**;
